# High-Throughput Sequencing—The Key to Rapid Biodiversity Assessment of Marine Metazoa?

**DOI:** 10.1371/journal.pone.0140342

**Published:** 2015-10-19

**Authors:** Inga Mohrbeck, Michael J. Raupach, Pedro Martínez Arbizu, Thomas Knebelsberger, Silke Laakmann

**Affiliations:** Department German Center for Marine Biodiversity Research, Senckenberg am Meer, Wilhelmshaven, Germany; University of Veterinary Medicine Hanover, GERMANY

## Abstract

The applications of traditional morphological and molecular methods for species identification are greatly restricted by processing speed and on a regional or greater scale are generally considered unfeasible. In this context, high-throughput sequencing, or metagenetics, has been proposed as an efficient tool to document biodiversity. Here we evaluated the effectiveness of 454 pyrosequencing in marine metazoan community analysis using the 18S rDNA: V1-V2 region. Multiplex pyrosequencing of the V1-V2 region was used to analyze two pooled samples of DNA, one comprising 118 and the other 37 morphologically identified species, and one natural sample taken directly from a North Sea zooplankton community. A DNA reference library comprising all species represented in the pooled samples was created by Sanger sequencing, and this was then used to determine the optimal similarity threshold for species delineation. The optimal threshold was found at 99% species similarity, with 85% identification success. Pyrosequencing was able to identify between fewer species: 67% and 78% of the species in the two pooled samples. Also, a large number of sequences for three species that were not included in the pooled samples were amplified by pyrosequencing, suggesting preferential amplification of some genotypes and the sensitivity of this approach to even low levels of contamination. Conversely, metagenetic analysis of the natural zooplankton sample identified many more species (particularly gelatinous zooplankton and meroplankton) than morphological analysis of a formalin-fixed sample from the same sampling site, suggesting an increased level of taxonomic resolution with pyrosequencing. The study demonstrated that, based on the V1-V2 region, 454 sequencing does not provide accurate species differentiation and reliable taxonomic classification, as it is required in most biodiversity monitoring. The analysis of artificially prepared samples indicated that species detection in pyrosequencing datasets is complicated by potential PCR-based biases and that the V1-V2 marker is poorly resolved for some taxa.

## Introduction

In light of an estimated half a million marine species, of which one- to two- thirds may be undescribed [1], biodiversity assessment remains one of our greatest challenges. Besides limited knowledge of how many and which species exist, it is not clearly predictable how anthropogenic forces and global environmental change affect marine biota and thus alter its biodiversity. Unquestionably, biodiversity research and ocean conservation planning require accurate species inventories and continuous monitoring [2]. Traditionally, species composition has been determined by morphological identification, reliant on specialized taxonomic expertise. The scarcity of professional taxonomists for some marine taxa often results in identifications to higher taxonomic units and an insufficient knowledge of biodiversity (e.g. [3,4]). Also, major difficulties arise in the morphological recognition of cryptic species and where individuals within a species exhibit phenotypic plasticity [5–7]. Furthermore, the correct identification of planktonic larvae and juveniles of fish and marine invertebrates to species or genus level, as well as the presence of damaged specimens in environmental samples, is particularly problematic in ecological studies [8,9]. To overcome these problems associated with classical species identification methods, faster and more precise molecular-based approaches are increasingly being tested, established and applied [10–14]. The build-up of public sequence databases, such as GenBank (www.ncbi.nlm.nih.gov) [15] and more specifically the Barcode of Life Data System (www.barcodinglife.org) [16] provides the basis for a DNA-based molecular identification system using DNA sequences, which can be assigned to already described and identified species [17]. This approach allows an accurate identification of metazoan specimens, eggs, larvae and even fragments of organisms [18,19]. For animals, mitochondrial DNA (mtDNA) exhibits several characteristics that make it highly attractive for molecular discrimination and identification [20–25], such as generally high substitution rates, almost exclusively maternal inheritance and lack of recombination [22,26–29]. Here, a fragment of approximately 650 base-pairs (bp) from the mitochondrial cytochrome *c* oxidase subunit 1 (CO1) gene was proposed as a global standard for molecular species identification of animals, the so-called “DNA barcode” [10]. DNA barcoding relies on low levels of mtDNA variation within species in combination with clear genetic differentiation between species. However, drawbacks of using CO1, and mtDNA in general, for species identification, include the possible co-amplification of nuclear mitochondrial pseudogenes (numts) [30,31], introgression through hybridization or incomplete lineage sorting [32–34], and heteroplasmy [35]. Therefore, supplementary nuclear markers, such as the complete 18S rDNA or its hypervariable expansion segments (V4, V7), segments of 28S rDNA (D1-D2, D3-D5), and internal transcribed spacers (ITS1, ITS2, 5.8S rDNA), have been used for species identification in several studies [3,20,22,36–40]. It should be noted that the issues listed here for mtDNA can also be associated with nuclear markers, for example intragenomic variations among rRNA gene copies and the occurrence of rDNA pseudogenes. However, only a few cases of intragenomic variations have been observed for metazoans [41–45]: Multiple variants of the SSU gene have been detected in a dinoflagellate [46], a platyhelminth [47] and sturgeons [48,49].

The classic approach to DNA sequence analysis generally involves amplification of DNA from single specimens and Sanger chain termination sequencing [50]. Next-generation sequencing (NGS) technologies on the other hand allow a comparably rapid and cost-effective high-throughput production of sequence data by amplification of environmental samples containing DNA from whole communities [51–53]. In this context, 454 pyrosequencing [54,55] has become a widely used NGS technique for various biological applications, as it provides long read lengths in comparison to other platforms and therefore facilitates taxonomic assignment [56]. Also DNA can be directly extracted from environmental samples (eDNA), reducing sample processing effort and the time required to isolate target specimens. Current 454 sequencing applications focus on metagenomic studies of uncultured microorganisms [57–60], meiofaunal and eukaryotic metagenetic community analyses [61–64], environmental barcoding linked to a given reference database [65,66] and metabarcoding of very short DNA fragments. These latter metabarcoding methods may not provide full taxonomic resolution, but can be used even if DNA is fragmented or degraded [67–71]. For most metazoan taxa, CO1 sequence data are highly abundant in public reference libraries, however, many studies have used nuclear ribosomal markers for high-throughput analysis of metazoan community composition [61–63,72–76]. This is owing to poor conservation of CO1 primers or primer regions across some major taxonomic groups, including decapod and peracarid crustaceans, resulting in an inconsistent amplification of some representatives of these taxa. Additionally, CO1 primers generally target the amplification of the complete barcode region [77], which is too large for current NGS platforms (read length was limited to about 450 bp at the outset of this project, and GS FLX Titanium chemistry had reached up to 800 bp by project end). In this context, smaller CO1 fragments (< 400 bp) amplified by newly designed primers have been quite effective for species identification of various metazoan taxa (e.g. [65,78–80]). Nevertheless, successful species identification based on CO1 increases with sequence length: species resolution for full-length DNA barcodes is 97%, whilst for barcodes of 100 bp it is only 90% [79]. The hypervariable V1-V2 expansion segments of 18S rDNA represent an alternative for molecular species identification. The V1-V2 region has already been applied as a molecular marker in metagenetic analyses of meiofauna and zooplankton assemblages [62,63,72,81] and was also applied in the present study. With a size of ~ 450 bp, this fragment can be easily sequenced through next-generation platforms such as the Roche Genome Sequencer (GS) FLX. Moreover, highly conserved rDNA regions flank fast-evolving regions, which has facilitated the design of universal primers that enable successful amplification across most of the major eukaryote taxa [39,82,83]—a necessity for the assessment of overall sequence abundance and taxa composition in environmental samples.

Although only a few published studies have focused on high-throughput DNA-based identifications of metazoans [61–63,65,66,70–72,74–76,80], 454 sequencing has been proposed as a fast and efficient tool for rapid and increasingly automated biodiversity assessment that also includes environmental metabarcoding. In fact, the efficiency of the NGS-based approach as a molecular identification tool to assess eukaryotic species richness is still under-explored and remains subject to discussion. The analysis of millions of sequence data generated from an environmental sample is one of the major challenges facing biologists. Moreover, accurate species identification is complicated by different error types related to inaccuracies in the sequencing process (e.g. base under- and over-calls associated with presence and size of homopolymers) [84,85] and PCR amplification steps prior to pyrosequencing. Putative PCR amplification errors account for 4–25% of the total and can present a crucial error source in high throughput environmental analyses [86], thus a robust understanding of these error types is essential. PCR amplification bias and artifact formation (e.g. chimeras generated during PCR amplification) can result in a large amount of missing data or an overestimation of diversity and may lead to a misinterpretation of species composition [87–89]. Given the significance of valid species identification for ecosystem biodiversity studies, quality assessment and validation of sequence data generated by NGS sequencers is of critical importance.

The aim of this study was to evaluate the potential application of 454 pyrosequencing, with respect to its reliability and accuracy, in species identification. To provide a valid and reliable basis for downstream analysis of the 454 data, we first constructed an 18S rDNA: V1-V2 reference library of 118 North Sea species (verified by both morphological identification and CO1 barcodes) using Sanger sequencing. The chosen gene region was further evaluated for its effectiveness in species identification by clustering at different sequence similarity thresholds. Multiplex pyrosequencing of the V1-V2 region was then used to analyze replicates of two samples containing DNA pooled from the previously Sanger-sequenced species, and one natural sample from a North Sea zooplankton community using the 454 GS FLX Titanium platform (Roche 454 Life Sciences, Branford, USA). The 454-derived sequences were compared to our Sanger reference library, using the clustering program cd-hit-est-2d [90], to test if the generated sequence reads would represent all of the species included in the pooled samples.

## Material and Methods

### Ethics Statement

No permit was needed to conduct the sampling and research was carried out on unprotected and non-endangered species only. All specimen collections were performed in accordance with institutional and national laws and ethical principles.

### Construction of the 18S rDNA: V1-V2 reference library

#### DNA extraction

In order to construct an 18S rDNA: V1-V2 reference library, we used DNA extracts from 118 North Sea species from a wide range of taxonomic groups encompassing ten metazoan phyla: Phoronida (one species), Ctenophora (one species), Cnidaria (one species), Chaetognatha (one species), Mollusca (17 species), Annelida (five species), Echinodermata (five species), Chordata (subphylum Tunicata, one species; subphylum Vertebrata, 17 species), and Arthropoda (subphylum Crustacea, 69 species) (Table 1, see also S1 Table for more information on target species). Each species was represented by a single individual, which was morphologically identified to species level by a taxonomic expert, except for three individuals (Cyclopoida sp., *Pomatoschistus* sp. and Spatangidea sp., see S1 Table). A single leg or tissue sample was then subjected to DNA extraction using the Qiagen DNEasy blood & tissue extraction kit (Hilden, Germany) following the manufacturer’s instructions. DNA concentrations were quantified fluorometrically using the Qubit dsDNA BR Assay Kit (Invitrogen, Life Technologies Europe, Naerum, Denmark). All measurements were made using 1 μl of DNA added to 199 μl of assay buffer prepared as directed by the manufacturer. The 200 μl samples were then transferred to Qubit Assay Tubes and measured with the Qubit 3.0 Fluorometer.

**Table 1:** Higher taxa composition and number of different species per taxa analyzed for the Sanger-sequence reference library used in the pooled DNA samples.

(Sub-) Phylum	(Sub-) Class	No. of species per taxa
Phoronida	-	1
Ctenophora	Tentaculata	1
Cnidaria	Scyphozoa	1
Chaetognatha	Sagittoidea	1
Mollusca	Bivalvia	7
	Gastropoda	5
	Scaphopoda	1
	Cephalopoda	4
Annelida	Polychaeta	5
Echinodermata	Ophiuroidea	2
	Asteroidea	2
	Echinoidea	1
Tunicata	Appendicularia	1
Crustacea	Thecostraca	3
	Copepoda	15
	Branchiopoda	3
	Pentastomida	1
	Malacostraca	47
Vertebrata	Actinopterygii	17

#### PCR amplification and Sanger sequencing

Morphological identifications were cross-referenced using CO1 barcodes; fragments were amplified following standard protocols [77] and sequenced using the Sanger sequencing method [50]. DNA barcodes were then compared to the authors’ own as well as publicly available sequence data, specifically on the Barcode of Life Data System (BOLD) webpage [16]. The hypervariable V1-V2 region (~ 450 bp) of the nSSU marker was amplified using the metazoan-specific primers SSU_F04 and SSU_R22 [63,91]. Polymerase chain reactions (PCR) were performed in 25 μl volumes using Illustra PureTaq PCR beads from GE Healthcare Life Science (Buckinghamshire, UK). PCRs contained 1 μl of each primer (100 pmol/μl) and 3 μl of DNA template. Amplification was conducted using an Eppendorf Mastercycler pro S thermocycler (Hamburg, Germany) with the following parameters: initial denaturation at 95°C for 2 minutes followed by 35 cycles repeating the sequence of 95°C for 1 minute, 57°C for 45 seconds and 72°C for 3 minutes. Final extension was performed at 72°C for 10 minutes. PCR products were confirmed by size with electrophoresis on a 1% agarose gel with GelRed (Biotium, Hayward, USA) using DNA size standards. The remaining PCR product was purified using the QIAquick PCR purification kit (Qiagen, Hilden, Germany). All purified PCR products were outsourced for Sanger sequencing to a contract sequencing facility (Macrogen, Seoul, Korea) using the same primer sets as for PCR.

#### Treatment of Sanger-generated sequences and determination of the optimal sequence similarity threshold

Sequences were processed using the software Geneious version 7.1.9 (Biomatters, Ltd. available from www.geneious.com) [92]. All new sequences were compared with those available in GenBank by BLAST searches [93,94] and subsequently deposited in the GenBank data library (accession numbers are presented in S1 Table). The clustering software cd-hit-est [90] was used to define an optimal threshold for species delineation of the V1-V2 region. Cd-hit first sorts sequences in decreasing length order, whereby the longest sequence becomes the representative of the first cluster. Then, each sequence is compared pairwise to this first sequence using a pre-defined sequence similarity threshold. If the similarity of a sequence pairing is equal to or higher than the given threshold, the new sequence is assigned to the first cluster, otherwise a new cluster is defined by this next sequence. The predefined threshold for the analysis was 0.98 or 98% similarity. In order to allow a direct comparison of the quality of filtered 454 data with the Sanger-generated sequences, the effect of homopolymer length reduction on taxonomic resolution in our reference library was investigated. Firstly the length of the longest homopolymers (6-mer, representing all occurrences of AAAAAA, TTTTTT, CCCCCC and GGGGGG) present in the Sanger reference library was determined. Then all detected 6-mer homopolymers were reduced by 1 nucleotide, resulting in 5-mer homopolymers (representing all occurrences of AAAAA, TTTTT, CCCCC and GGGGG). This step was repeated three more times by reducing homopolymer length from n = 5 to n = 4 (AAAA, TTTT, CCCC, GGGG), n = 4 to n = 3 (AAA, TTT, CCC, GGG), and n = 3 to n = 2 (AA, TT, CC, GG). Altogether four reference libraries were produced in this way, characterized by 5-mer, 4-mer, 3-mer and 2-mer homopolymers, respectively. Finally cluster analysis of each library was performed at 97, 98, 99 and 100% similarity thresholds using the program cd-hit-est [90]

### Sample pooling and sample preparation for 454 pyrosequencing

Two artificial samples were prepared by pooling DNA extracts (5 μl each) from the Sanger-sequenced species. The first sample, hereafter referred to as ALLDNA, contained DNA from all 118 Sanger-sequenced species, including both pelagic and benthic organisms. The second sample, hereafter referred to as ZPDNA, was composed of extracts from 37 of the 118 species, specifically holo- and meroplanktonic organisms (S1 Table). In addition, a natural community of zooplankton species was analyzed. For the natural community sample, two quantitative zooplankton samples (500 μm mesh size) were successively collected at the Helgoland time series site (54°11.3`N, 7°54.0`E) on October 1^st^ 2010. One sample was fixed in formaldehyde-seawater (4%) for species identification using light microscopy analysis in the framework of the Helgoland Roads long-term plankton monitoring program [95]. Organisms present in this sample were identified to species or genus level where possible, but some taxa, in particular gelatinous zooplankton and meroplanktonic larvae (e.g. Polychaeta, Bivalvia) could only be identified to major taxonomic groups. The second sample was preserved in absolute ethanol for metagenetic analysis of the V1-V2 region. To extract genomic DNA from this bulk sample it was first washed over a 40 μm mesh sieve with distilled water. The resultant slurry was divided equally between fourteen 2 ml Eppendorf tubes, into each of which 720 μl ATL lysis buffer and 80 μl Proteinase K (Qiagen, Hilden, Germany) were added. The tubes were then incubated at 56°C at 400 rpm in an Eppendorf thermomixer (Hamburg, Germany) for three hours and subsequently centrifuged at 9000 g for 2 minutes. After this centrifugation step, the supernatant was transferred into new tubes and 400 μl of a buffer AL-ethanol mixture (Qiagen, Hilden, Germany) was added to each. DNA extraction was then performed using, and according to the manufacturer′s protocol for, the Qiagen DNEasy blood & tissue extraction kit. Finally, 20 μl aliquots from each of the 14 independently isolated DNA extracts were combined in one tube, resulting in a total volume of 280 μl. This sample is hereafter referred to as ZPHTS.

The V1-V2 region of the 18S rDNA was amplified from both the pooled DNA mixtures of 118 (ALLDNA) and 37 (ZPDNA) species and the ethanol-preserved natural zooplankton sample (ZPHTS) using the primer pair SSU_F04 and SSU_R22 [63,91]. Amplification reactions and cycling parameters were conducted under the same conditions as for the establishment of the Sanger-sequenced reference library, yielding ~ 450 bp products. After visualization of 2 μl of the PCR products on an agarose gel, all products were subsequently purified using QIAquick PCR Purification (Qiagen, Hilden, Germany) and then divided into two aliquots (70 μl each) per sample, which served as templates to construct the amplicon library.

### 454 pyrosequencing

Pyrosequencing of the V1-V2 region was performed by the company GATC Biotech (Constance, Germany). After ligation of 454 adapters and emulsion PCR (emPCR) amplification, using the forward primer F04 and a Phusion high-fidelity PCR master mix (New England Biolabs GmbH, Frankfurt am Main, Germany), the six tagged amplicons (two replicates per sample) were pooled in equal amounts and sequenced on 1/8 of a picotiter plate (70x75 cm) of the 454 GS FLX Titanium sequencer (Roche 454 Life Sciences, Branford, USA).

#### Data filtering

Raw reads obtained from pyrosequencing were processed with Geneious version 7.1.9 [92] as follows: Firstly, reads were discarded if they had (1) an average quality score < 10% of the sequence, (2) more than two mismatches in the primer sequence, or (3) < 200 bp. Adaptor and primer sequences were then trimmed from each read before subsequent analysis. Because 454 pyrosequencing is known to be particularly error-prone in homopolymeric regions (i.e. AAAAAAA), some further degree of quality filtering of the 454 data is required to minimize downstream artifacts. However, the task of removing homopolymers in particular is not straightforward and applied algorithms can result in over-trimmed reads and thus the loss of potentially informative data. Therefore, a manual approach of homopolymer error correction was tested that would still allow a direct comparison to the Sanger reference library. In each of the 454-derived sequences homopolymer repeats beyond 4-mers were reduced to three consecutive identical bases (3-mers). This value was determined by the Sanger reference library study of homopolymer length reduction and species-level resolution. Moreover, this 3-mers value also corresponds relatively well to the accuracy of base calling in a 454 run. For example, a typical 454 sequencing run can determine a single base with an accuracy of 99% for 3-base homopolymers, but only 64% accuracy for 9-mers due to an increased variability in signal intensity [96]. Consequently, cluster analyses were conducted of the 3-mers quality-filtered 454 data against the 3-mers Sanger reference library at a similarity threshold of 99%.

#### Cluster analysis

All quality-filtered 454 reads were compared against the 3-mers reference library at 99% sequence similarity using the program cd-hit-est-2d [90] at default settings. Reads from the natural zooplankton community sample (ZPHTS) that did not correspond to those in the reference library were subjected to a second cluster analysis using cd-hit-est at a 99% similarity level, low coverage clusters (< 10 reads) then removed. For clusters, or molecular operational taxonomic units (MOTUs) exhibiting ≥ 10 reads, MEGABLAST [93] searches were performed against the GenBank database. If score and similarity match were greater than 100 and 90%, respectively, sequences were assigned to the best possible taxonomic rank, i.e. the species with the highest score in the result list. If multiple species had the same level of nucleotide similarity to a sequence then it was assigned to the lowest, shared taxonomic level. Statistical analysis was carried out using R 2.14.2 (R Development Core Team; www.r-project.org/). Spearman’s rank correlation tests were performed to examine the reproducibility of pyrosequencing by comparing read distribution of each aliquot and to determine whether the number of reads was dependent on DNA concentration or DNA GC-content. All correlation analyses were conducted by using the function “cor.test” and the method = “spearman”.

## Results

### Determination of the optimal sequence similarity threshold for species delineation

Cluster analysis of the Sanger-generated reference library showed that the proposed V1-V2 region separated 75% (88 species) of the 118 species tested at a sequence similarity threshold of 98% (Table 2). The remaining 30 species formed clusters that contained two or more species. Species-level resolution improved with increased clustering stringency: At a 99% similarity threshold about 85% of sequences could be resolved to individual species. Separate species could not be resolved for 18 sequences: eight species had 99% similarity to other congenerics in the 18S rDNA sequence (genera *Idotea*, *Liocarcinus*, *Pagurus* and *Ophiura*) and seven species had 99% sequence similarity to a member of the same order (Decapoda). A similarity threshold of 100% greatly increased the number of observed clusters represented by one species each, but still failed to differentiate all of the 118 species: One cluster contained three confamiliar species (*Pleuronectes platessa*, *Limanda limanda*, *Kareius bicoloratus*, family Pleuronectidae) which showed no sequence variation in the V1-V2 18S rDNA fragment.

**Table 2:** Species delineation results for the Sanger-generated reference library at a 98% similarity threshold. For each cluster comprising more than one sequence a representative sequence* is presented.

Cluster-No.	MT-No.	Species ID	Similarity threshold (%)
0	MT1161	*Clistosaccus paguri*	98
1	MT0001	*Jassa marmorata*	98
2	MT1110	*Monocorophium insidiosum*	98
3	MT0024	*Jassa falcata*	98
4	MT1240	*Caprella mutica*	98
5	MT0795	*Caligus elongatus*	98
6	MT1278	*Reighardia lomviae*	98
7	MT0742	*Eudorella truncatula*	98
8	MT0814	*Euterpina acutifrons*	98
9	MT0064	*Diogenes pugilator*	98
10	MT0008	*Echinogammarus marinus*	98
11	MT1231	*Semibalanus balanoides*	98
12	MT1155*	*Idotea granulosa*	
	MT0070	*Idothea balthica*	98
	MT1156	*Idothea pelagica*	99
13	MT1050	*Loligo forbesi*	98
14	MT0779*	*Pseudocalanus elongatus*	98
15	MT0680	*Evadne nordmanni*	98
16	MT1127	*Monocorophium sextonae*	98
17	MT0094	*Idotea linearis*	98
18	MT1435	*Phoronis muelleri*	98
19	MT1140	*Janira maculosa*	98
20	MT0584*	*Centropages typicus*	
	MT0541	*Centropages hamatus*	98
21	MT0598	*Ditrichocorycaeus anglicus*	98
22	MT1039	*Ensis directus*	98
23	MT1251*	*Ebalia cranchii*	
	MT1256	*Liocarcinus depurator*	99
	MT1255	*Liocarcinus navigator*	99
	MT1298	*Carcinus maenas*	98
	MT1254	*Liocarcinus marmoreus*	98
	MT1462	*Hemigrapsus takanoi*	99
	MT1162	*Goneplax rhomboides*	98
24	MT1266	*Macropodia parva*	98
25	MT0998	*Nucula nitidosa*	98
26	MT1217	*Galathea dispersa*	98
27	MT1250	*Pandalus montagui*	98
28	MT0793	Cyclopoida *sp*.	98
29	MT1198	*Philocheras bispinosus*	98
30	MT0948	*Aequipecten opercularis*	98
31	MT1377	*Oithona similis*	98
32	MT1413	*Oikopleura (Vexillaria) dioica*	98
33	MT1176	*Jaera (Jaera) albifrons*	98
34	MT0160*	*Pagurus prideaux*	
	MT0170	*Pagurus pubescens*	99
35	MT0497	*Temora longicornis*	98
36	MT1363	*Podon intermedius*	98
37	MT1023	*Antalis entalis*	98
38	MT1601*	*Neptunea antiqua*	
	MT0983	*Colus gracilis*	98
39	MT1423	*Sabellaria alveolata*	98
40	MT0078	*Upogebia deltaura*	98
41	MT1599	*Phaxas pelludicus*	98
42	MT0216	*Asterias rubens*	98
43	MT1436	Spatangidea *sp*.	98
44	MT1259	*Galathea intermedia*	98
45	MT1036	*Mactra stultorum*	98
46	MT0820	*Parasagitta setosa*	98
47	MT1146	*Praunus flexuosus*	98
48	MT0532	*Paraeuchaeta norvegica*	98
49	MT1590	*Sepiola atlantica*	98
50	MT1018*	*Aporrhais pespelicani*	
	MT0974	*Buccinum undatum*	98
51	MT1330	*Neomysis integer*	98
52	MT1216	*Balanus crenatus*	98
53	MT0150*	*Ophiura ophiura*	
	MT0194	*Ophiura sarsii*	99
54	MT1325	*Magelona* cf. *mirabiis*	98
55	MT0564	*Calanus helgolandicus*	98
56	MT1177	*Homarus gammarus*	98
57	MT1390	*Lagis koreni*	98
58	MT1241*	*Corystes cassivelaunus*	
	MT0087	*Cancer pagurus*	99
	MT1281	*Hyas araneus*	99
59	MT0797	*Tomopteris (Johnstonella) helgolandica*	98
60	MT1594	*Chamelea gallina*	98
61	MT1589	*Todaropsis eblanae*	98
62	MT0180	*Luidia sarsi*	98
63	MT0857*	*Melanogrammus aeglefinus*	
	MT0283	*Gadus morhua*	98
64	MT0620	*Myoxocephalus scorpius*	98
65	MT0627	*Trisopterus esmarkii*	98
66	MT0792	*Pleurobrachia pileus*	98
67	MT1260	*Hyas coarctatus*	98
68	MT0617	*Buglossidium luteum*	98
69	MT0649	*Argentina sphyranea*	98
70	MT0323	*Crystallogobius linearis*	98
71	MT0641	*Hippoglossoides platessoides*	98
72	MT0075	*Idotea emarginata*	98
73	MT0051	*Hyperia galba*	98
74	MT1364	*Podon leuckartii*	98
75	MT0750	*Lanice conchilega*	98
76	MT1277	*Ligia oceanica*	98
77	MT1049	*Loligo vulgaris*	98
78	MT1164	*Epimeria cornigera*	98
79	MT1188	*Monocorophium acherusicum*	98
80	MT0605	*Arnoglossus laterna*	98
81	MT0902*	*Pleuronectes platessa*	
	MT0761	*Limanda limanda*	100
	MT1252	*Kareius bicoloratus*	100
82	MT0273	*Clupea harengus*	98
83	MT0264*	*Trisopterus minutus*	
	MT0874	*Merluccius merluccius*	99
84	MT0217	*Aurelia aurita*	98
85	MT0522	*Anomalocera patersoni*	98
86	MT0487	*Trachurus trachurus*	98
87	MT1128	*Apherusa bispinosa*	98
88	MT1133	*Orchestia mediterranea*	98
89	MT0293	*Pomatoschistus sp*.	98
90	MT1003	*Euspira catena*	98
91	MT1207	*Spirontocaris lilljeborgii*	98
92	MT0082	*Crangon crangon*	98
93	MT0055	*Mytilus edulis*	98
94	MT0737	*Monopsudocuma gilsoni*	98
95	MT0830	*Paracalanus parvus*	98
96	MT0735	*Pseudocuma (Pseudocuma) simile*	98
97	MT0833	*Acartia (Acartiura) clausi*	98
98	MT0556	*Acartia (Acanthacartia) tonsa*	98

**Fig 1:**
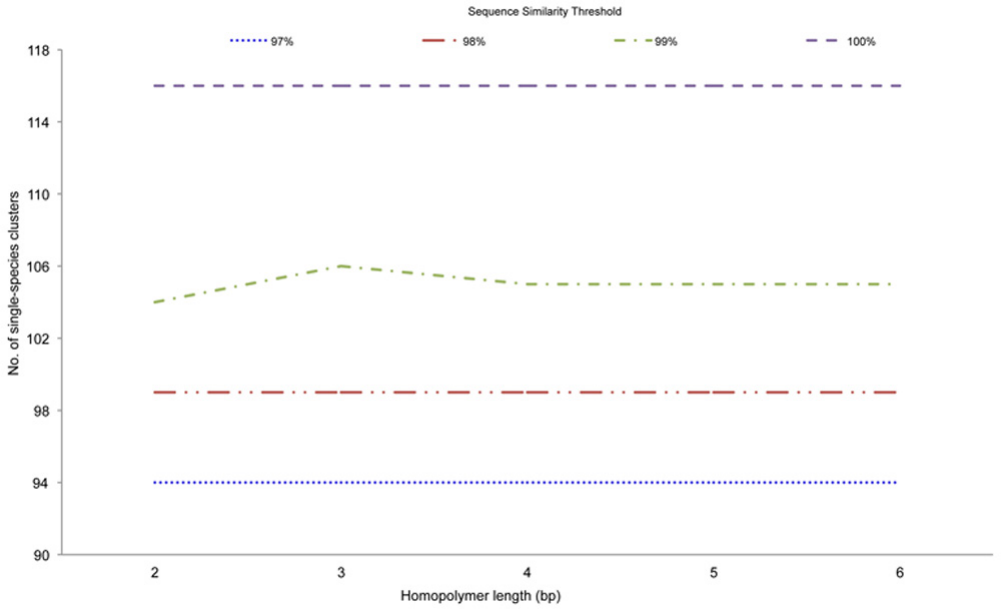
Cluster analysis conducted on modified reference libraries (characterized by 5-mer, 4-mer, 3-mer and 2-mer homopolymers, respectively) using different similarity thresholds (97–100%, at 1% intervals).

In order to investigate the effect of homopolymer length reduction on species level resolution, we also performed cluster analyses on modified reference libraries (characterized by 5-mer, 4-mer, 3-mer and 2-mer homopolymers, respectively) using different similarity thresholds (97–100%, at 1% intervals). At a 99% similarity threshold, the number of clusters increased from 105 to 106 if homopolymer length was reduced to three identical nucleotides (Fig 1) and decreased to 104 clusters if homopolymer length was reduced to two identical nucleotides. At all other thresholds, homopolymer length reduction made no effect on species level resolution (the number of clusters remained constant). Thus, subsequent analyses were performed using a high-similarity sequence identity of 99% with homopolymers reduced to three consecutive identical nucleotides (3-mers).

### 454 pyrosequencing results

The total number of 454 derived sequences was as follows: from 118 pooled species in the ALLDNA sample, 55,264 and 50,883 sequences (aliquots 1 and 2, respectively); from 37 pooled species in the ZPDNA sample, 54,558 (aliquot 1) and 52,288 sequences (aliquot 2); and from the natural zooplankton sample ZPHTS, 49,807 and 52,762 sequences (aliquot 1 and 2, respectively). The 454 datasets have been deposited in GenBank under the study accession number SRP035575 (run accession numbers SRR1138374 and SRR1138375, SRR1141384 and SRR1141385, SRR1138366 and SRR1138371, see Table 3). Quality filtering (i.e. removal of sequences with an average quality score < 10, with > 2 mismatches in the primer sequence or with < 200 bp length) reduced the total number of sequences for ALLDNA to 31,014 and 29,891 (aliquots 1 and 2, respectively), to 42,324 and 38,639 for ZPDNA aliquots 1 and 2, respectively, and for ZPHTS aliquots 1 and 2 to 34,044 and 36,097, respectively. Pyrosequencing reads of the 118 (ALLDNA) and 37 (ZPDNA) pooled DNA extracts and the field-collected zooplankton sample (ZPHTS) were then used as queries against our Sanger-generated reference library of 118 North Sea metazoans at 99% sequence similarity threshold. A neighbor-joining tree based on uncorrected p-distances from all reference sequences was constructed using Mega 5.2.2 [97] in order to visualize the percentage of reads for each species detected from each sample by pyrosequencing.

**Table 3:** GS FLX sequences generated for each aliquot.

Sample aliquot	No. of reads (raw data)	Min	Mean	Max	Std. dev.	GenBank accession no.
**ALLDNA1**	55264	36	298.1	804	141.4	SRR1138374
**ALLDNA2**	50882	36	307.2	550	135.8	SRR1138375
**ZPDNA1**	54558	36	359.0	1187	95.6	SRR1141384
**ZPDNA2**	52288	36	343.5	636	113.8	SRR1141385
**ZPHTS1**	49807	36	333.9	723	124.7	SRR1138366
**ZPHTS2**	52762	36	334.0	660	124.7	SRR1138371

#### Read distribution of pooled, known DNA samples

**Fig 2:**
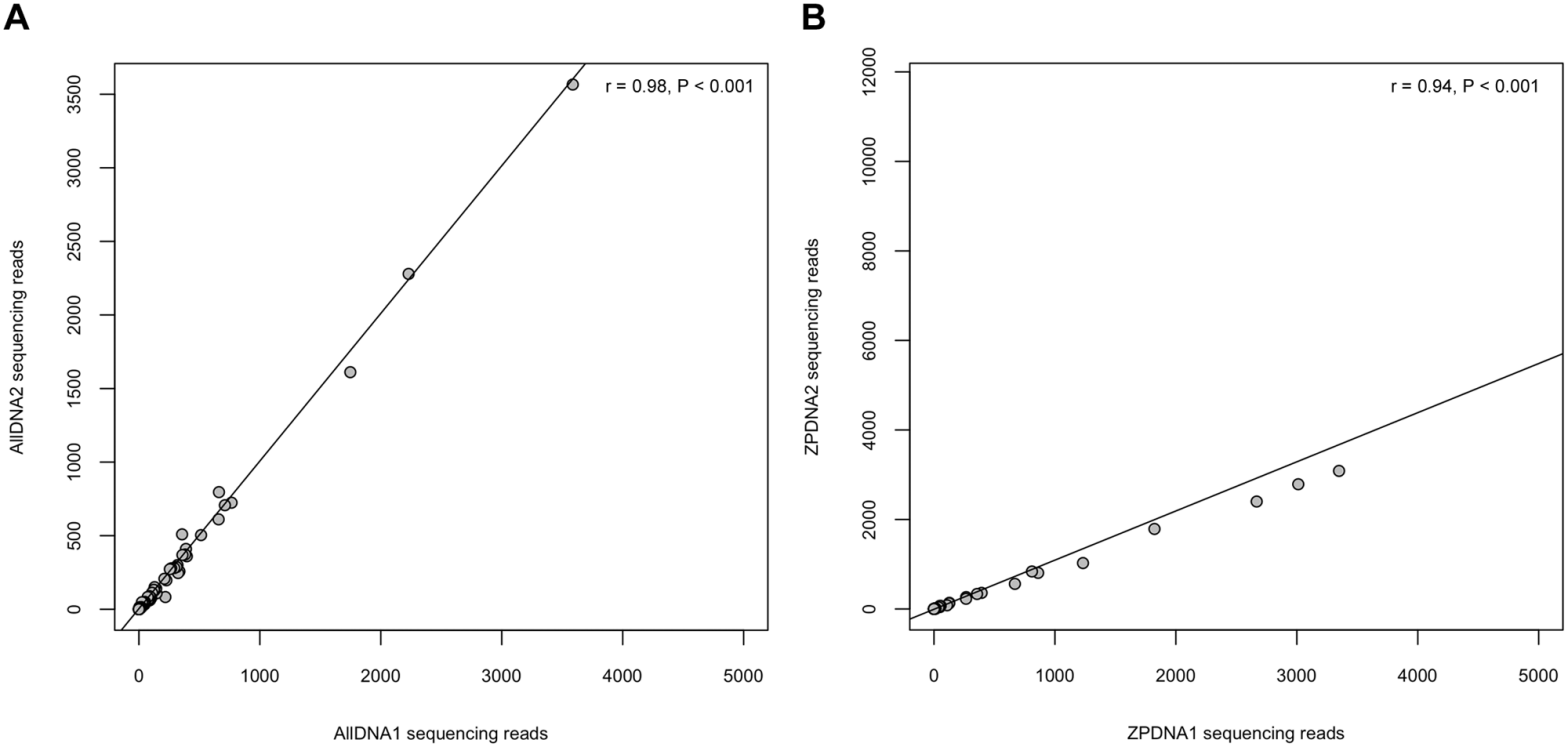
Read coverage distribution of sample aliquots inferred from pyrosequencing after clustering against the reference library at 99% similarity. **(A)** Dataset showing strong positive correlation between aliquot 1 (*x*-axis) and aliquot 2 (*y*-axis) of pooled ALLDNA sample (Spearman r = 0.98, P < 0.001). (**B)** Dataset showing strong positive correlation between aliquot 1 (*x*-axis) and aliquot 2 (*y*-axis) of pooled ZPDNA sample (Spearman r = 0.94, P < 0.001).

**Fig 3:**
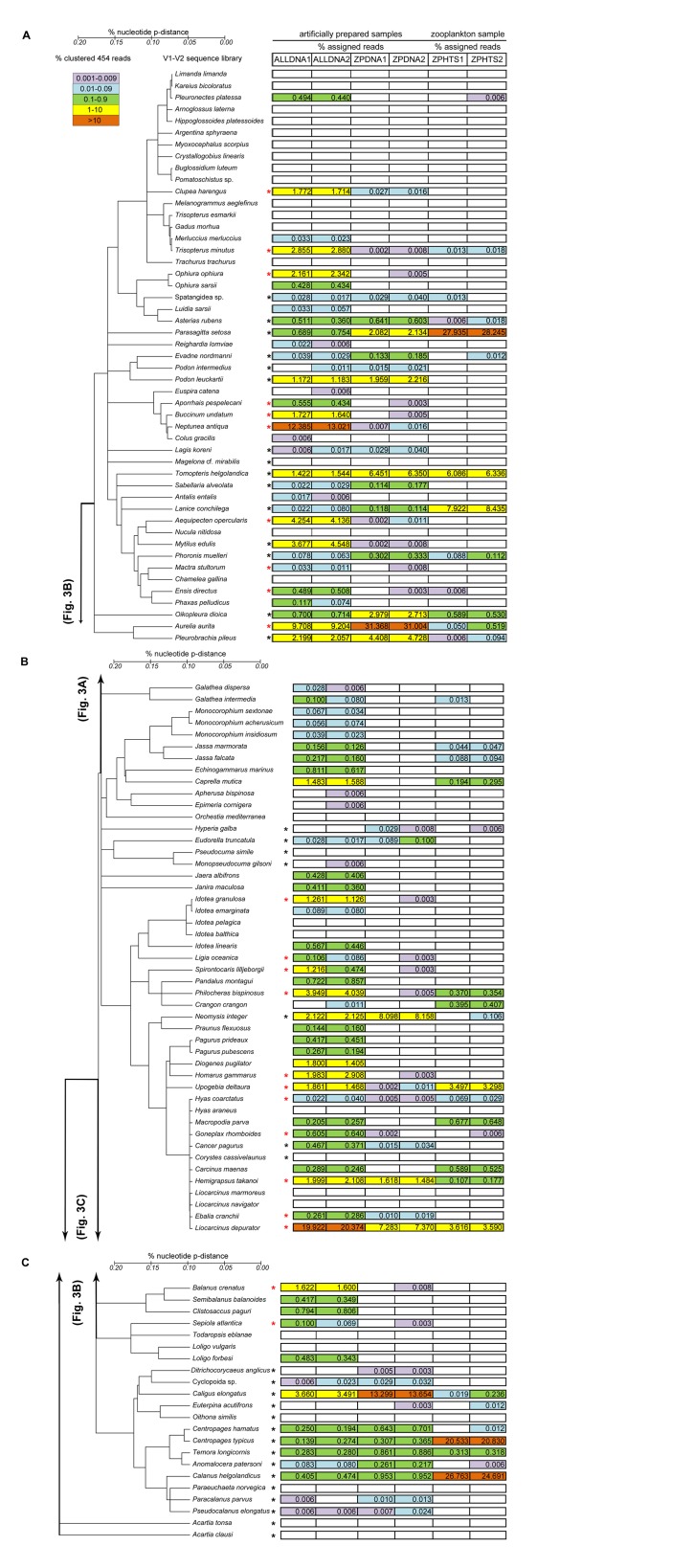
A) Species composition of North Sea metazoa from four aliquots of pooled, known DNA samples (ALLDNA, ZPDNA) and two aliquots of one field-collected zooplankton sample (ZPHTS), as assigned by 454 sequencing of the 18S rDNA: V1-V2 region. The tree, based on consensus sequences of the Sanger-generated reference library, was generated by the Neighbor-Joining algorithm / p-distance model. Clustering against the reference library was performed at 99% sequence similarity threshold using the program cd-hit-est-2d. Percentage of pyrosequencing reads for each species detected in each sample is shown by color-coded rectangles. Black asterisks denote species included in the artificially prepared ZPDNA sample. Red asterisks denote species not included, but detected in the ZPDNA sample. **B) Figure 3A continued. C) Figure 3B continued**.

Clustering of ALLDNA aliquots against Sanger sequences generated a total number of 18,005 (58%) and 17,503 (59%) clustered reads for aliquots 1 and 2, respectively. There was a strong significant correlation between aliquots 1 and 2 of the ALLDNA sample (Fig 2A), since the same species had the highest read numbers (Spearman correlation coefficient (r) = 0.98, P < 0.001). Of the 118 species that were originally pooled in the ALLDNA sample 79 (67% for aliquot 1) and 83 (70% for aliquot 2) were detected by pyrosequencing (Fig 3). The majority of missing species were representatives of the taxa Crustacea and the Vertebrata (Actinopterygii). The percentage of undetected species was highest in Actinopterygii at about 76% for both sample aliquots.

Clustering of the ZPDNA sample aliquots against our reference library produced 34,821 (82%) and 32,071 (83%) clustered reads for aliquots 1 and 2, respectively. The number of reads per cluster were relatively similar between the two aliquots and significantly correlated (r = 0.94, P < 0.001) (Fig 2B). Reads could be assigned to 40 (aliquot 1) and 53 (aliquot 2) species of the reference library (Fig 3). However, the ZPDNA sample comprised only 37 species extracts (Fig 3, black asterisks) and of these 28 (76% for aliquot 1) and 29 (78% for aliquot 2) species were recorded. The species included in the ZPDNA sample but not detected by clustering (eight species in aliquot 1 and seven in aliquot 2), were, except for one polychaete species (*Magelona* cf. *mirabilis*), all Crustacea (particularly Copepoda), which represented 65% of the pooled mixture. The clustering also recorded species (12 or 32% in aliquot 1 and 24 or 65% in aliquot 2), despite them not being present in the ZPDNA sample (Fig 3, red asterisks). Most of these species had a low number of reads (< 10), with the exception of *Aurelia aurita* (12973 and 11724 reads), *Liocarcinus depurator* (3012 and 2787 reads) and *Hemigrapsus takanoi* (669 and 561 reads) (Fig 3A and 3B). However, the same reference-based cluster analyses were also conducted on the original datasets (before homopolymer reduction) and, except for a lower fraction of assignable reads (between 1% and 7%), the results were congruous with those above (data not shown).

Spearman’s correlation coefficients (r), calculated between DNA concentrations of the extracts pooled and the number of pyrosequencing reads generated from each aliquot, demonstrated a very weak but significant correlation for both ALLDNA aliquots (r = 0.2, P = 0.03 for aliquot 1; r = 0.2, P ≤ 0.05 for aliquot 2), whilst there was no significant relationship observed for the ZPDNA aliquots (r = -0.08, P = 0.6 for aliquot 1; r = -0.09, P = 0.6 for aliquot 2) (see S1A and S1B Fig). Since most DNA polymerases used for PCR can introduce significant GC biases we also investigated whether the number of reads obtained for each species was dependent on the GC richness of the V1-V2 region (see S1C and S1D Fig). There were no significant correlations between the number of reads and the GC content of the pooled ALLDNA sample (r = -0.1, P = 0.3 and r = -0.14, P = 0.1, aliquot 1 and 2 respectively) (S1C Fig) and the pooled ZPDNA sample (r = -0.06, P = 0.7 and r = -0.07, P = 0.7, aliquot 1 and 2 respectively) (S1D Fig).

#### Read distribution of the field-collected zooplankton sample

For the metagenetic analysis of a natural zooplankton sample (ZPHTS) the read distribution was consistent with those for the pooled samples, being positively correlated (r = 0.84, P < 0.001) between the two aliquots ZPHTS1 and ZPHTS2 (data not shown). Reference-based clustering of the 454 sequences resulted in clustered reads of 15,955 (47%) and 16,966 (47%) for aliquots 1 and 2, respectively, and 27 and 32 distinct species, respectively, that matched a species in the reference library with a similarity ≥ 99% (Fig 3, zooplankton sample). These species included annelids, arthropods, chordates and polychaetes, as well as 11 taxa that were also found in the formalin-fixed sample collected concurrently and identified morphologically (Table 4). In terms of sequence numbers both metagenetic sample aliquots were dominated by copepods (46%) and the chaetognath *Parasagitta setosa* (28%) (Fig 3A). For copepods, read coverage was particularly high (>20%) for two calanoid species that were recorded, *Calanus helgolandicus* (4270 and 4189 reads) and *Centropages typicus* (3276 and 3534 reads) (Table 4, Fig 3C). *Calanus* and *Centropages* are common genera at Helgoland Roads and made up 18% of the total formalin-fixed sample (0.15 and 0.53 individuals/m^3^, respectively) (Table 4). Metagenetic analysis also revealed a large number of sequence records (14%) with a high degree of similarity (≥ 99%) to two polychaete species, *Lanice conchilega* and *Tomopteris helgolandica* (Fig 3A), which are commonly documented around Helgoland, but which were not found in the formalin-fixed sample. Meroplanktonic larvae of decapod species, in particular *Liocarcinus* spp. and *Upogebia deltaura* comprised about 9% of the sequence records in both ZPHTS aliquots (Fig 3B), whereas in the morphologically analyzed sample they amounted to 30% of total individuals. Both metagenetic and morphological methods found the genera *Liocarcinus* and *Upogebia* to dominate the meroplankton (Table 4).

**Table 4:** Comparison of taxonomic composition of a morphologically analyzed (individuals/m^3^) and a pyrosequenced zooplankton sample, as aliquots ZPHTS1 and ZPHTS2 (no. of reads per species). Identifications of the pyrosequenced sample determined by searching against a V1-V2 reference library (99% similarity) and the GenBank database (MEGABLAST search > 90% identity).

Morphologically analyzed zooplankton sample		Read coverage ZPHTS1		Read coverage ZPHTS2	
Taxonomic entities of holo- and meroplankton	Ind./m^3^	Matches from V1-V2 sequence library (99% cut-off)	Matches from GenBank (>90% identity)	Matches from V1-V2 sequence library (99% cut-off)	Matches from GenBank (>90% identity)
*Calanus* spp.	0.148	4270	650	4189	628
*Centropages typicus*	0.529	3276	382	3534	391
*Temora* spp.	0.037	56	no match	58	no match
*Acartia* spp.	0.006	no match	no match	no match	no match
*Pseudocalanus* spp.	0.012	no match	no match	no match	no match
*Mesopodopsis slabberi*	0.006	no reference sequence	no match	no reference sequence	no match
Gammaridae spp.	0.019	no match	no match	no match	no match
*Pantopoda* spp. larva	0.012	no reference sequence	219	no reference sequence	190
*Diastylis* spp.	0.006	no reference sequence	no match	no reference sequence	no match
*Crangon crangon* larva	0.08	63	211	69	173
*Eualus* spp. larva	0.012	no reference sequence	22	no reference sequence	22
*Pagurus bernhardus* larva	0.006	no reference sequence	no match	no reference sequence	no match
*Pisidia longicornis* larva	0.258	no reference sequence	no match	no reference sequence	no match
*Callianassa* spp. larva	0.006	no reference sequence	no match	no reference sequence	no match
*Upogebia* spp. larva	0.578	558	no match	558	no match
*Carcinus maenas* larva	0.006	94	16	89	17
*Liocarcinus* spp. larva	0.135	577	no match	609	no match
*Macropodia rostrata* larva	0.012	no reference sequence	no match	no reference sequence	no match
Leptothecata spp.	0.031	no reference sequence	1612	no reference sequence	1670
*Eucheilota maculata*	0.006	no reference sequence	1085	no reference sequence	1096
*Mitrocomella polydiademata*	0.16	no reference sequence	no match	no reference sequence	no match
*Obelia* spp.	0.006	no reference sequence	15	no reference sequence	no match
*Clytia hemisphaerica*	0.572	no reference sequence	2389	no reference sequence	3007
Polychaeta	0.431	971	390	1075	379
Syllidae spp.	0.012	no reference sequence	no match	no reference sequence	no match
*Parasagitta setosa*	0.228	4457	383	4792	412
Fish eggs	0.043	2	21	4	22
Bivalvia larva	0.006	1	no match	no match	no match
*Penilia avirostris*	0.314	no reference sequence	312	no reference sequence	275
Ophiuroidae spp. pluteus	0.006	no match	41	no match	39
*Oikopleura dioica*	0.012	94	no match	90	no match

**Fig 4:**
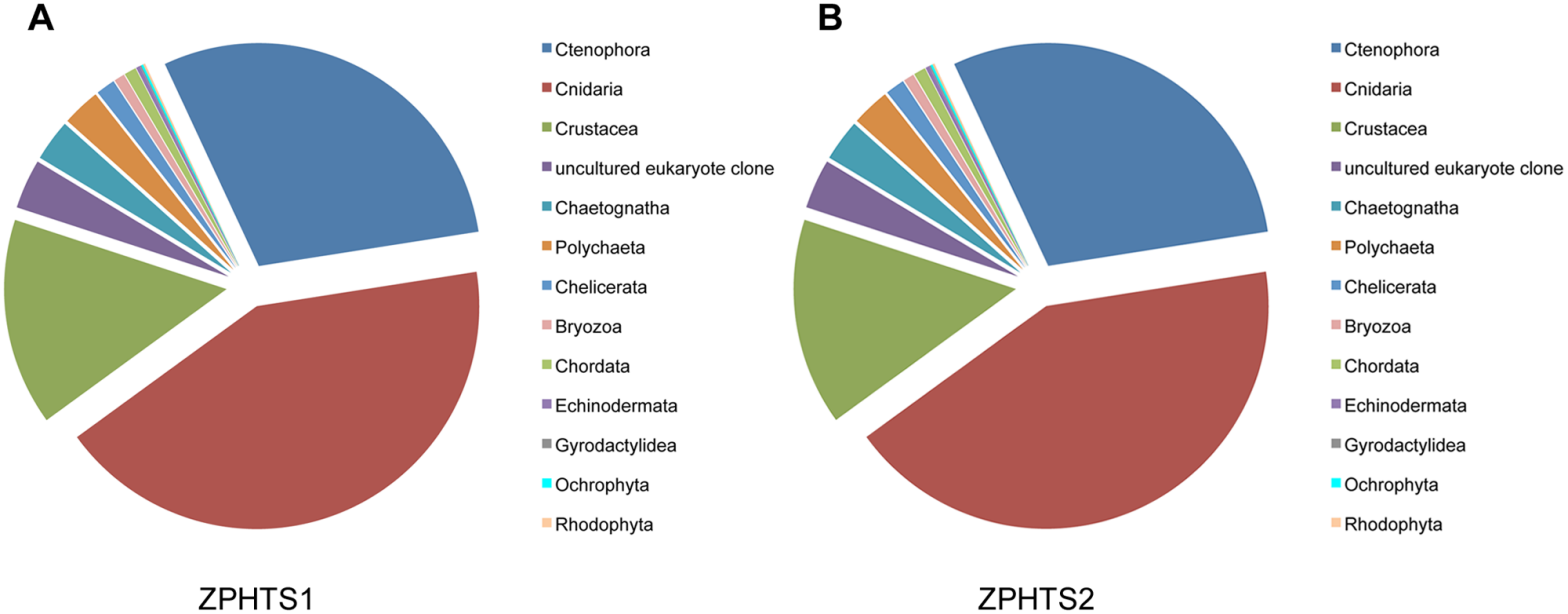
Pie chart illustrating the relative proportion of each phylum detected by MEGABLAST analysis (score and sequence similarity > 100 and 90%, respectively). **(A)** ZPHTS aliquot 1. **(B)** ZPHTS aliquot 2. MEGABLAST hits to ‘uncultured eukaryote clone’ represent unclassified taxa.

To determine the identity of those reads that were not assignable to a species in the reference library (about 53%) a second cluster analysis was conducted at 99% similarity threshold. Removal of low coverage clusters (<10 reads) resulted in 155 and 157 clusters, or MOTUs, for aliquots 1 and 2, respectively. Representative sequences of each MOTU were then subjected to MEGABLAST analyses against the GenBank database. Several of the sequences provided multiple hits for different but closely related species and at the same level of nucleotide similarity, for example, the copepod species *Calanus helgolandicus* and *Calanus pacificus* (both with 98.2% similarity), the brown shrimp *Crangon crangon* and the grass shrimp *C*. *franciscorum* (98% similarity), and several other decapod species, including *Necora puber* and *Homalaspis plana* (98% similarity). In such cases, the name of the hierarchically lowest, shared taxonomic grouping was assigned to the given sequence record, for example, the sequence matching both *C*. *helgolandicus* and *C*. *pacificus* was named *Calanus* sp. Representatives of the Cnidaria (e.g. the hydrozoans *Clytia hemisphaerica*, *Plumularia setacea* and *Eucheilota maculata*) and Ctenophora (e.g. *Beroe* sp.) were identified as most highly represented in both sample aliquots ranging from 30% to 40% of the total sequences, followed by the Crustacea (e.g. *Schistomysis kervillei* (Mysida), *Penilia avirostris* (Branchiopoda) and Calanoida sp. (Copepoda)) that made up to 15% of the sequence records (Fig 4). In comparison, hydrozoans, in particular *Clytia hemisphaerica*, were also highly abundant in the formalin-fixed sample (21% of individuals, or 0.8 individuals/m^3^), whereas ctenophores were not recorded (see Table 4). The MEGABLAST search also matched a small number of sequences to the phyla Ochrophyta (a diatom species of the genus *Odontella* with 19 and 20 reads) and Rhodophyta (*Delesseria* sp. with 19 and 20 reads) (Fig 4). However, these taxa are not considered in macrozooplankton morphological analyses and thus were not analyzed in the formalin-fixed sample. Interestingly, our NGS analysis also revealed the presence of the monogenean parasitic genus *Gyrodactylus* (11 reads) that was also not identified in the formalin-fixed sample. In total, 176 (aliquot 1) and 188 (aliquot 2) species were estimated for the zooplankton sample using metagenetic methods, but for the formalin-fixed sample only 31 species of holoplankton (permanent inhabitants of the planktonic community) and meroplankton (larvae of benthic invertebrates and fish) were identified by morphological analysis.

## Discussion

The application of 454 pyrosequencing has become quite attractive in biodiversity research due to its potential utility in analyzing complex communities on a large scale. The major goal of this study was to evaluate the potential and feasibility of 454 pyrosequencing in marine metazoan community analysis and applied biodiversity assessment studies. Here, a comprehensive reference sequence library was constructed of 118 North Sea species based on Sanger sequencing of the V1-V2 region of the 18S rDNA gene. To directly compare DNA-based identifications of 454 sequencing with traditional identification methods, the V1-V2 region was pyrosequenced from replicates of two pooled samples containing DNA from the 118 previously Sanger sequenced species. In addition, the species composition of a natural zooplankton community was analyzed to compare species richness estimated by metagenetic and morphological methods.

### The optimal similarity threshold for species identification

In a threshold and cluster-based approach, as performed in this study, taxonomic units are usually identified using an empirical similarity threshold. Consequently, the choice of a certain threshold value can considerably affect species identification accuracy and lead to an under- or overestimation of biodiversity [98]. Thus to provide a reliable basis for downstream analysis of the 454 data, we first determined an optimal similarity threshold for the V1-V2 region of the 18S rDNA gene. The reference library produced in this study allowed the investigation of taxonomic resolution in the V1-V2 region across a wide range of taxonomic groups by testing a range of similarity cut-off values. Trials showed that if the similarity threshold was increased from 97% to 98% the number of species clusters increased from 93 to 98, a 5% increase, and if similarity was further increased to 99% the number of clusters rose to 104, an additional 6% increase. Overall, therefore, it was indicated that the V1-V2 region is a good molecular marker for species-level identifications for most of the metazoans analyzed here, although a 100% similarity threshold would still have failed to identify three confamiliar species (*Pleuronectes platessa*, *Limanda limanda* and *Kareius bicoloratus*, Table 2). Compared to mtCO1, the 18S rDNA gene is characterized by a much slower sequence evolution, potentially making it a more suitable marker for taxonomic classification at higher ranks such as family and above [99]. Nevertheless, the V1-V2 expansion segments analyzed here show a high variability in their primary sequence and length even between closely related species as a consequence of DNA slippage-like processes (e.g. [100–102]) which typically allow a discrimination of even closely related species (e.g. [22,103–106]).

### Reliability and accuracy of 454 sequencing in species identification

The effectiveness in species identification was investigated by comparing replicates of two artificial samples of pooled DNA extracts and one field-collected zooplankton sample. The robustness and reproducibility of 454 sequencing in the present study was generally high with sample replicates showing only little variation between runs. However, pyrosequencing was able to identify only between 67% and 78% of the species used to constitute the pooled mixtures. Interestingly, some species (e.g. *Acartia clausi*, *Paraeuchaeta norvegica*, *Pseudocuma simile* and *Chamelea gallina*) were not detected in the entire pyrosequencing experiment (Fig 3), but were accurately separated in the threshold-based analysis conducted on the Sanger-generated reference library. Recent studies have suggested that species represented by singular small individuals and low frequency species can be missed by 454 sequencing [65,66], but this is unlikely to explain the present results. In this study DNA from a single individual per species was used for sample preparation and considerable differences were found in the number of sequences generated for each of the species. Moreover, we observed a high read coverage for some small individuals (e.g. the copepod species *Caligus elongatus*, body length ~ 4 mm), whilst some of the larger species (e.g. the mysid *Neomysis integer*, body length ~ 15 mm) had lower read coverage or were even absent (e.g. the bivalve *Chamelea gallina*, length ~ 35 mm) in both 454 sequenced samples. However, nuclear rDNAs are generally organized in tandem repeats with hundreds of copies [107,108], potentially facilitating a high read coverage of some species independent of size and/or biomass. Previous work has found that the GC content may have a strong effect on PCR amplification and amplicon based 454-sequencing [109–111]. To that end, the efficiency in sequencing GC-rich templates and its impact on species detection was examined. However, no significant correlations between read coverage biases and variation in GC-content were observed for any of the analyzed datasets. In addition, read coverage of each cluster was correlated only very weakly or not at all with the DNA concentration of that species, suggesting that this bias was introduced during PCR amplification rather than by differences in DNA concentration or rDNA copy number. Since the 454 approach applied here involves two different PCR amplification steps (amplification during initial sample preparation and emulsion PCR on the 454 sequencing platform), PCR biases such as differential amplification efficiencies, can significantly affect the representation (i.e. the presence or absence of species) of a sampled community. However, for certain taxa (particularly Actinopterygii) the failure in identification is more likely to result from insufficient taxonomic resolution of the SSU marker. In fact, several studies have already recommended a multiple-marker-approach (e.g. [66,76]) and have shown that the use of two markers significantly enhances the level of species detection. Although multi-marker community analysis excludes problems associated with using a single marker, such as the effects of heteroplasmy, incomplete lineage sorting for mitochondrial markers, and missing resolution in the case of nuclear rDNAs, the weaknesses of 454-based species identification clearly correlate with PCR bias and homopolymer reading errors. Therefore, the use of specific bidirectional primers for 454 sequencing of multiple genes, ideally linked to a comprehensive reference library of Sanger sequenced specimens, may be an opportunity to mitigate PCR biases and further improve species detection, more than the use of biological replicates [66]. However, the inclusion of supplementary markers and the use of both forward and reverse sequences will not only improve species detection but also increase the cost, which is an issue when performing routine NGS-based species identifications in monitoring programs.

It is of concern that pyrosequencing not only failed to identify all species pooled in the test samples, but 'detected' species that were not present in these samples (12 and 24 species in ZPDNA aliquots 1 and 2, respectively). For the majority of these sequences there was a low coverage (>10 reads), with the exception of three species (*Aurelia aurita*, *Liocarcinus depurator* and *Hemigrapsus takanoi*), which exhibited high read numbers in both sample aliquots. Carew et al. [66] suggested that the threshold for accepting the presence of a species should be set at five or more sequences per sample. In our study, even the application of a > 10 reads threshold would exclude 8 and 21 (aliquot 1 and 2, respectively) of these “unexpected” sequence records, but also eliminate 7 species that were originally included in the pooled sample. Other examples of unexpected sequence records from 454 analyses, have been assuming to result from ethanol-based carry-over of DNA to specimens that were stored in the same preservation liquid prior DNA extraction [65]. In this study, the presence of high read coverage for “contaminants” such as *Aurelia aurita* suggests the sensitivity of the 454 method and indicates the preferential amplification of some loci even with only modest contamination.

### Metagenetic and morphological diversity of a natural zooplankton community

The metagenetic analysis of the natural community of zooplankton collected at Helgoland Roads revealed a substantially higher diversity than the morphological analysis of the formalin-fixed sample from the same sampling site. However, it should be noted that this is mainly to the inclusion of meroplanktonic larvae and some Copepoda, particularly their early stages, which are difficult to identify to species or genus level through microscopic examination. As a result there are only a few, in some cases only one, putative species or grouping(s) defined for certain morphologically similar groups (e.g. bivalves, polychaetes, fish eggs and larvae), whereas high-throughput analysis identified several species for each of these groups. Thus in this case high-throughput analysis had a higher taxonomic resolution than morphological analysis. About one fifth (24%) of the species identified in the metagenetic sample, were also recorded in the morphological sample, indicating that these species were actually collected. The large number of sequences found for the two polychaete species *T*. *helgolandica* and *L*. *conchilega* in the metagenetic sample, was supported by their known seasonal phenology (see also www.senckenberg.de/dzmb/plankton) [95], despite their absence from the morphological sample. MEGABLAST search provided multiple hits for different, but closely related, species and with the same level of nucleotide similarity. As already discussed above, the V1-V2 region of the 18S rDNA may not offer enough information for precise taxonomic classification and the use of more rapidly evolving loci, in particular the CO1 barcode fragment, may be more effective to distinguish closely related species. However, the assessment of overall sequence abundances and taxa composition in environmental samples requires the design of universal primers to enable successful amplification across eukaryote groups.

### Conclusions

The study has demonstrated that various issues have to be resolved until metagenetic methods can be routinely used for automated biodiversity assessment in environmental samples. The inclusion of artificially prepared samples showed that accurate species detection from pyrosequencing datasets was limited by the poor resolution of the 18S rDNA gene region for certain taxa and also by PCR-based biases. Therefore, extreme caution should be used when inferring relative species abundances from pyrosequencing data. Efficient monitoring of species diversity requires the accurate differentiation of species and information on spatial and temporal variations in species abundance and dominance. When sequencing accuracy becomes critical, 454 pyrosequencing may not be the right choice: Quantity does not replace quality.

## Supporting Information

S1 Fig Scatter plot demonstrating correlations between DNA concentration and GC richness of extracts pooled and number of assigned pyrosequencing reads generated for each aliquot by clustering against Sanger reference library. (TIF)

S1 Table Information on Sanger sequenced specimens used for marker analysis and preparation of bulk mixtures. (DOCX)
