# Supplementary figures and images for: High-Throughput Sequencing—The Key to Rapid Biodiversity Assessment of Marine Metazoa?
Source: PLoS One. 2015 Oct 19;10(10):e0140342. doi: 10.1371/journal.pone.0140342 (PMC4610693; doi:10.1371/journal.pone.0140342)

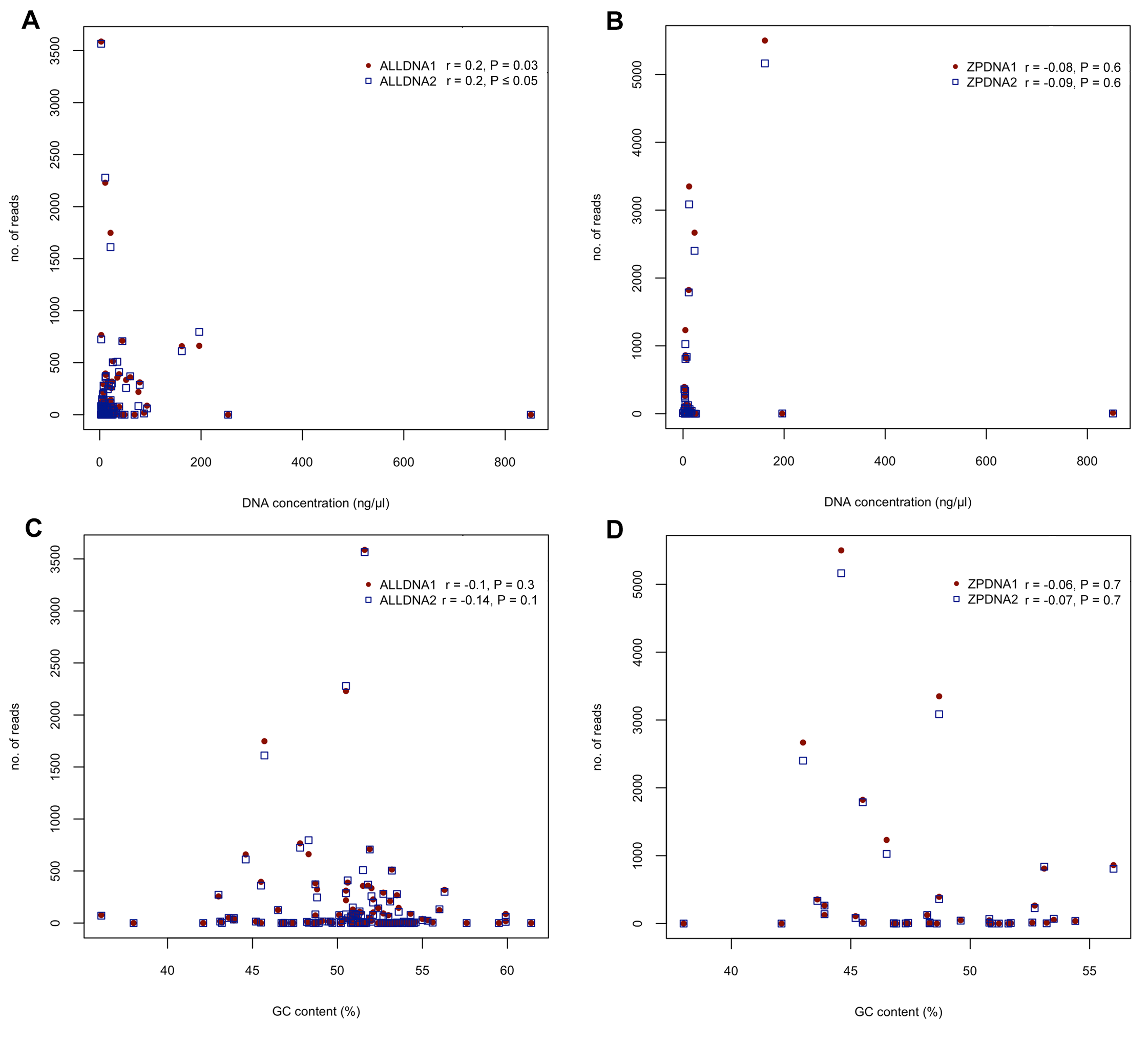

Supplement: S1 Fig — (TIF) [file pone.0140342.s001.tif]
