# Supplementary material for: High-Throughput Sequencing—The Key to Rapid Biodiversity Assessment of Marine Metazoa?
Source: PLoS One. 2015 Oct 19;10(10):e0140342. doi: 10.1371/journal.pone.0140342 (PMC4610693; doi:10.1371/journal.pone.0140342)
Supplement: S1 Table — (DOCX) [file pone.0140342.s002.docx]

**Supporting information S1 Information on Sanger sequenced specimens utilized for marker analysis and preparation of bulk mixtures.**

| **MT-No.** | **(Sub-) Phylum** | **(Sub-) Class** | **(Sub-) Order** | **Family** | **Species** | **Fixation** | **GenBank accession numbers** | **AllDNA** | **ZPDNA** |
| --- | --- | --- | --- | --- | --- | --- | --- | --- | --- |
|  |  |  |  |  |  |  | **18S rDNA: V1-V2** |  |  |
|  |  |  |  |  |  |  |  |  |  |
| 1435 | Phoronida | - | - | - | *Phoronis muelleri* Selys-Lonchamps, 1903 | 96% EtOH | KJ193748 | X | X |
| 0792 | Ctenophora | Tentaculata | Cydippida | Pleurobrachiidae | *Pleurobrachia pileus* (O. F. Müller, 1776) | 96% EtOH | KJ193811 | X | X |
| 0217 | Cnidaria | Scyphozoa | Semaeostomeae | Ulmaridae | *Aurelia aurita* (Linnaeus, 1758) | 96% EtOH | KJ193834 | X |  |
| 0820 | Chaetognatha | Sagittoidea | Aphragmophora | Sagittidae | *Parasagitta setosa* (Müller, 1847) | 96% EtOH | KJ193782 | X | X |
| 1599 | Mollusca | Bivalvia | Euheterodonta | Pharidae | *Phaxas pellucidus* (Pennant, 1777) | 96% EtOH | KJ193776 | X |  |
| 1039 | Mollusca | Bivalvia | Euheterodonta | Pharidae | *Ensis directus* (Conrad, 1843) | 96% EtOH | KJ193753 | X |  |
| 1594 | Mollusca | Bivalvia | Veneroida | Veneridae | *Chamelea gallina* (Linnaeus, 1758) | 96% EtOH | KJ193805 | X |  |
| 1036 | Mollusca | Bivalvia | Veneroida | Mactridae | *Mactra stultorum* (Linnaeus, 1758) | 96% EtOH | KJ193781 | X |  |
| 0998 | Mollusca | Bivalvia | Nuculida | Nuculidae | *Nucula nitidosa* Winckworth, 1930 | 96% EtOH | KJ193756 | X |  |
| 0948 | Mollusca | Bivalvia | Pectinoida | Pectinidae | *Aequipecten opercularis* (Linnaeus, 1758) | 96% EtOH | KJ193764 | X |  |
| 0055 | Mollusca | Bivalvia | Mytiloida | Mytilidae | *Mytilus edulis* Linnaeus, 1758 | 96% EtOH | KJ193842 | X | X |
| 0974 | Mollusca | Gastropoda | Neogastropoda | Buccinidae | *Buccinum undatum* Linnaeus, 1758 | 96% EtOH | KJ193803 | X |  |
| 0983 | Mollusca | Gastropoda | Neogastropoda | Buccinidae | *Colus gracilis* (da Costa, 1778) | 96% EtOH | KJ193789 | X |  |
| 1601 | Mollusca | Gastropoda | Neogastropoda | Buccinidae | *Neptunea antiqua* (Linnaeus, 1758) | 96% EtOH | KJ193773 | X |  |
| 1003 | Mollusca | Gastropoda | Littorinimorpha | Naticidae | *Euspira catena* (da Costa, 1778) | 96% EtOH | KJ193839 | X |  |
| 1018 | Mollusca | Gastropoda | Littorinimorpha | Aporrhaidae | *Aporrhais pespelicani* (Linnaeus, 1758) | 96% EtOH | KJ193786 | X |  |
| 1023 | Mollusca | Scaphopoda | Dentaliida | Dentaliidae | *Antalis entalis* (Linnaeus, 1758) | 96% EtOH | KJ193772 | X |  |
| 1049 | Mollusca | Cephalopoda | Myopsida | Loliginidae | *Loligo vulgaris* Lamarck, 1798 | 96% EtOH | KJ193823 | X |  |
| 1050 | Mollusca | Cephalopoda | Myopsida | Loliginidae | *Loligo forbesi* Steenstrup, 1856 | 96% EtOH | KJ193743 | X |  |
| 1589 | Mollusca | Cephalopoda | Oegopsida | Ommastrephidae | *Todaropsis eblanae* (Ball, 1841) | 96% EtOH | KJ193806 | X |  |
| 1590 | Mollusca | Cephalopoda | Sepiolida | Sepiolidae | *Sepiola atlantica* d'Orbigny, 1839 | 96% EtOH | KJ193785 | X |  |
| 0750 | Annelida | Polychaeta | Terebellida | Terebellidae | *Lanice conchilega* (Pallas, 1766) | 96% EtOH | KJ193821 | X | X |
| 1390 | Annelida | Polychaeta | Terebellida | Pectinariidae | *Lagis koreni* Malmgren, 1866 | 96% EtOH | KJ193797 | X | X |
| 1325 | Annelida | Polychaeta | Spionida | Magelonidae | *Magelona* cf. *mirabilis* (Johnston, 1865) | 96% EtOH | KJ193794 | X | X |
| 1423 | Annelida | Polychaeta | Sabellida | Sabellariidae | *Sabellaria alveolata* (Linnaeus, 1767) | 96% EtOH | KJ193774 | X | X |
| 0797 | Annelida | Polychaeta | Phyllodocida | Tomopteridae | *Tomopteris (Johnstonella) helgolandica* (Greeff, 1879) | 96% EtOH | KJ193802 | X | X |
| 0150 | Echinodermata | Ophiuroidea | Ophiurida | Ophiuridae | *Ophiura ophiura* (Linnaeus, 1758) | 96% EtOH | KJ193793 | X |  |
| 0194 | Echinodermata | Ophiuroidea | Ophiurida | Ophiuridae | *Ophiura sarsii* Lütken, 1855 | 96% EtOH | KJ193801 | X |  |
| 0180 | Echinodermata | Asteroidea | Paxillosida | Luidiidae | *Luidia sarsi* Düben & Koren, 1845 | 96% EtOH | KJ193807 | X |  |
| 0216 | Echinodermata | Asteroidea | Forcipulatida | Asteriidae | *Asterias ruben*s Linnaeus, 1758 (larva) | 96% EtOH | KJ193777 | X | X |
| 1436 | Echinodermata | Echinoidea | Spatangoida | - | Spatangidea sp. Fischer, 1966 (larva) | 96% EtOH | KJ193778 | X | X |
| 1413 | Tunicata | Appendicularia | Copelata | Oikopleuridae | *Oikopleura (Vexillaria) dioica* Fol, 1872 | 96% EtOH | KJ193766 | X | X |
| 1161 | Crustacea | Maxillopoda | Akentrogonida | Clistosaccidae | *Clistosaccus paguri* Lilljeborg, 1860 | 96% EtOH | KJ193730 | X |  |
| 1231 | Crustacea | Maxillopoda | Sessilia | Archaeobalanidae | *Semibalanus balanoides* (Linnaeus, 1758) | 96% EtOH | KJ193741 | X |  |
| 1216 | Crustacea | Maxillopoda | Sessilia | Balanidae | *Balanus crenatus* Bruguiére, 1789 | 96% EtOH | KJ193791 | X |  |
| 0833 | Crustacea | Maxillopoda | Calanoida | Acartiidae | *Acartia (Acartiura) clausi* Giesbrecht, 1889 | 96% EtOH | KJ193846 | X | X |
| 0556 | Crustacea | Maxillopoda | Calanoida | Acartiidae | *Acartia (Acanthacartia) tonsa* Dana, 1849 | 96% EtOH | KJ193847 | X | X |
| 0564 | Crustacea | Maxillopoda | Calanoida | Calanidae | *Calanus helgolandicus* (Claus, 1863) | 96% EtOH | KJ193795 | X | X |
| 0541 | Crustacea | Maxillopoda | Calanoida | Centropagidae | *Centropages hamatu*s (Lilljeborg, 1853) | 96% EtOH | KJ193751 | X | X |
| 0584 | Crustacea | Maxillopoda | Calanoida | Centropagidae | *Centropages typicus* Krøyer, 1849 | 96% EtOH | KJ193750 | X | X |
| 0779 | Crustacea | Maxillopoda | Calanoida | Clausocalanidae | *Pseudocalanus elongatus* (Boeck, 1865) | 96% EtOH | KJ193744 | X | X |
| 0532 | Crustacea | Maxillopoda | Calanoida | Euchaetidae | *Paraeuchaeta norvegica* (Boeck, 1872) | 96% EtOH | KJ193784 | X | X |
| 0830 | Crustacea | Maxillopoda | Calanoida | Paracalanidae | *Paracalanus parvus* (Claus, 1863) | 96% EtOH | KJ193844 | X | X |
| 0497 | Crustacea | Maxillopoda | Calanoida | Temoridae | *Temora longicornis* (Müller O.F., 1785) | 96% EtOH | KJ193769 | X | X |
| 0522 | Crustacea | Maxillopoda | Calanoida | Pontellidae | *Anomalocera patersoni* Templeton, 1837 | 96% EtOH | KJ193835 | X | X |
| 0793 | Crustacea | Maxillopoda | Cyclopoida | - | Cyclopoida sp. Burmeister, 1835 | 96% EtOH | KJ193760 | X | X |
| 1377 | Crustacea | Maxillopoda | Cyclopoida | Oithonidae | *Oithona similis* Claus, 1866 | 96% EtOH | KJ193765 | X | X |
| 0814 | Crustacea | Maxillopoda | Harpacticoida | Euterpinidae | *Euterpina acutifrons* (Dana, 1847) | 96% EtOH | KJ193738 | X | X |
| 0598 | Crustacea | Maxillopoda | Poecilostomatoida | Corycaeidae | *Ditrichocorycaeus anglicus* (Lubbock, 1857) | 96% EtOH | KJ193752 | X | X |
| 0795 | Crustacea | Maxillopoda, | Siphonostomatoida | Caligidae | *Caligus elongatus* von Nordmann, 1832 | 96% EtOH | KJ193735 | X | X |
| 0680 | Crustacea | Branchiopoda | Cladocera | Podonidae | *Evadne nordmanni* Lovén, 1836 | 96% EtOH | KJ193745 | X | X |
| 1363 | Crustacea | Branchiopoda | Cladocera | Podonidae | *Podon intermedius* Lilljeborg, 1853 | 96% EtOH | KJ193771 | X | X |
| 1364 | Crustacea | Branchiopoda | Cladocera | Podonidae | *Podon leuckartii* (G.O. Sars, 1862) | 96% EtOH | KJ193820 | X | X |
| 1278 | Crustacea | Pentastomida | Cephalobaenida | Reighardiidae | *Reighardia lomviae* Dyck, 1975 | 96% EtOH | KJ193736 | X |  |
| 1128 | Crustacea | Malacostraca | Amphipoda | Calliopiidae | *Apherusa bispinosa* (Bate, 1857) | 96% EtOH | KJ193833 | X |  |
| 1240 | Crustacea | Malacostraca | Amphipoda | Caprellidae | *Caprella mutica* Schurin, 1935 | 96% EtOH | KJ193734 | X |  |
| 1188 | Crustacea | Malacostraca | Amphipoda | Corophiidae | *Monocorophium acherusicum* (Costa, 1853) | 96% EtOH | KJ193825 | X |  |
| 1110 | Crustacea | Malacostraca | Amphipoda | Corophiidae | *Monocorophium insidiosum* (Crawford, 1937) | 96% EtOH | KJ193732 | X |  |
| 1127 | Crustacea | Malacostraca | Amphipoda | Corophiidae | *Monocorophium sextonae* (Crawford, 1937) | 96% EtOH | KJ193746 | X |  |
| 1164 | Crustacea | Malacostraca | Amphipoda | Epimeriidae | *Epimeria cornigera* (Fabricius, 1779) | 96% EtOH | KJ193824 | X |  |
| 0008 | Crustacea | Malacostraca | Amphipoda | Gammaridae | *Echinogammarus marinus* (Leach, 1815) | 96% EtOH | KJ193740 | X |  |
| 0051 | Crustacea | Malacostraca | Amphipoda | Hyperiidae | *Hyperia galba* (Montagu, 1815) | 96% EtOH | KJ193819 | X | X |
| 0024 | Crustacea | Malacostraca | Amphipoda | Ischyroceridae | *Jassa falcata* (Montagu, 1808) | 96% EtOH | KJ193733 | X |  |
| 0001 | Crustacea | Malacostraca | Amphipoda | Ischyroceridae | *Jassa marmorata* Holmes, 1905 | 96% EtOH | KJ193731 | X |  |
| 1133 | Crustacea | Malacostraca | Amphipoda | Talitridae | *Orchestia mediterranea* Costa, 1853 | 96% EtOH | KJ193837 | X |  |
| 0742 | Crustacea | Malacostraca | Cumacea | Leuconidae | *Eudorella truncatula* (Bate, 1856) | 96% EtOH | KJ193737 | X | X |
| 0735 | Crustacea | Malacostraca | Cumacea | Pseudocumatidae | *Pseudocuma (Pseudocuma) simile* G.O. Sars, 1900 | 96% EtOH | KJ193845 | X | X |
| 0737 | Crustacea | Malacostraca | Cumacea | Pseudocumatidae | *Monopseudocuma gilsoni* (Gilson, 1906) | 96% EtOH | KJ193843 | X | X |
| 0087 | Crustacea | Malacostraca | Decapoda | Cancridae | *Cancer pagurus* Linnaeus, 1758 | 96% EtOH | KJ193800 | X | X |
| 1241 | Crustacea | Malacostraca | Decapoda | Corystidae | *Corystes cassivelaunus* (Pennant, 1777) (larva) | 96% EtOH | KJ193799 | X | X |
| 0082 | Crustacea | Malacostraca | Decapoda | Crangonidae | *Crangon crangon* (Linnaeus, 1758) | 96% EtOH | KJ193841 | X |  |
| 1198 | Crustacea | Malacostraca | Decapoda | Crangonidae | *Philocheras bispinosus* (Hailstone, 1835a) | 96% EtOH | KJ193761 | X |  |
| 0064 | Crustacea | Malacostraca | Decapoda | Diogenidae | *Diogenes pugilator* (Roux, 1829) | 96% EtOH | KJ193739 | X |  |
| 1217 | Crustacea | Malacostraca | Decapoda | Galatheidae | *Galathea dispersa* Bate, 1859 | 96% EtOH | KJ193757 | X |  |
| 1259 | Crustacea | Malacostraca | Decapoda | Galatheidae | *Galathea intermedia* Liljeborg, 1851 | 96% EtOH | KJ193779 | X |  |
| 1162 | Crustacea | Malacostraca | Decapoda | Goneplacidae | *Goneplax rhomboides* (Linnaeus, 1758) | 96% EtOH | KJ193792 | X |  |
| 1207 | Crustacea | Malacostraca | Decapoda | Hippolytidae | *Spirontocaris lilljeborgii* (Danielssen, 1859) | 96% EtOH | KJ193840 | X |  |
| 1266 | Crustacea | Malacostraca | Decapoda | Inachidae | *Macropodia parva* Van Noort & Adema, 1985 | 96% EtOH | KJ193755 | X |  |
| 1251 | Crustacea | Malacostraca | Decapoda | Leucosiidae | *Ebalia cranchii* Leach, 1817 | 96% EtOH | KJ193754 | X |  |
| 1177 | Crustacea | Malacostraca | Decapoda | Nephropidae | *Homarus gammarus* (Linnaeus, 1758) | 96% EtOH | KJ193796 | X |  |
| 1260 | Crustacea | Malacostraca | Decapoda | Oregoniidae | *Hyas coarctatus* Leach, 1816 | 96% EtOH | KJ193813 | X |  |
| 1281 | Crustacea | Malacostraca | Decapoda | Oregoniidae | *Hyas araneus* (Linnaeus, 1758) | 96% EtOH | KJ193804 | X |  |
| 0160 | Crustacea | Malacostraca | Decapoda | Paguridae | *Pagurus prideaux* Leach, 1815 | 96% EtOH | KJ193768 | X |  |
| 0170 | Crustacea | Malacostraca | Decapoda | Paguridae | *Pagurus pubescens* Krøyer, 1838 | 96% EtOH | KJ193798 | X |  |
| 1250 | Crustacea | Malacostraca | Decapoda | Pandalidae | *Pandalus montagui* Leach, 1814 | 96% EtOH | KJ193758 | X |  |
| 1254 | Crustacea | Malacostraca | Decapoda | Polybiidae | *Liocarcinus marmoreus* (Leach, 1814) | 96% EtOH | KJ193787 | X |  |
| 1255 | Crustacea | Malacostraca | Decapoda | Polybiidae | *Liocarcinus navigator* (Herbst, 1794) | 96% EtOH | KJ193762 | X |  |
| 1256 | Crustacea | Malacostraca | Decapoda | Polybiidae | *Liocarcinus depurator* (Linnaeus, 1758) | 96% EtOH | KJ193759 | X |  |
| 1298 | Crustacea | Malacostraca | Decapoda | Portunidae | *Carcinus maenas* (Linnaeus, 1758) | 96% EtOH | KJ193780 | X |  |
| 0078 | Crustacea | Malacostraca | Decapoda | Upogebiidae | *Upogebia deltaura* (Leach, 1815) | 96% EtOH | KJ193775 | X |  |
| 1462 | Crustacea | Malacostraca | Decapoda | Varunidae | *Hemigrapsus takanoi* Asakura & Watanabe, 2005 | 96% EtOH | KJ193788 | X |  |
| 0070 | Crustacea | Malacostraca | Isopoda | Idoteidae | *Idotea balthica* (Pallas, 1772) | 96% EtOH | KJ193763 | X |  |
| 0075 | Crustacea | Malacostraca | Isopoda | Idoteidae | *Idotea emarginata* (Fabricius, 1793) | 96% EtOH | KJ193818 | X |  |
| 0094 | Crustacea | Malacostraca | Isopoda | Idoteidae | *Idotea linearis* (Linnaeus, 1766) | 96% EtOH | KJ193747 | X |  |
| 1155 | Crustacea | Malacostraca | Isopoda | Idoteidae | *Idotea granulosa* Rathke, 1843 | 96% EtOH | KJ193742 | X |  |
| 1156 | Crustacea | Malacostraca | Isopoda | Idoteidae | *Idotea pelagica* Leach, 1815 | 96% EtOH | KJ193770 | X |  |
| 1176 | Crustacea | Malacostraca | Isopoda | Janiridae | *Jaera (Jaera) albifrons* Leach, 1814 | 96% EtOH | KJ193767 | X |  |
| 1140 | Crustacea | Malacostraca | Isopoda | Janiridae | *Janira maculosa* Leach, 1814 | 96% EtOH | KJ193749 | X |  |
| 1277 | Crustacea | Malacostraca | Isopoda | Ligiidae | *Ligia oceanica* (Linnaeus, 1767) | 96% EtOH | KJ193822 | X |  |
| 1330 | Crustacea | Malacostraca | Mysida | Mysidae | *Neomysis integer* (Leach, 1814) | 96% EtOH | KJ193790 | X | X |
| 1146 | Crustacea | Malacostraca | Mysida | Mysidae | *Praunus flexuosus* (Müller, 1776) | 96% EtOH | KJ193783 | X |  |
| 0264 | Vertebrata | Actinopterygii | Gadiformes | Gadidae | *Trisopterus minutus* (Linnaeus, 1758) | 96% EtOH | KJ193830 | X |  |
| 0627 | Vertebrata | Actinopterygii | Gadiformes | Gadidae | *Trisopterus esmarkii* (Nilsson, 1855) | 96% EtOH | KJ193810 | X |  |
| 0283 | Vertebrata | Actinopterygii | Gadiformes | Gadidae | *Gadus morhua* Linnaeus, 1758 | 96% EtOH | KJ193812 | X |  |
| 0857 | Vertebrata | Actinopterygii | Gadiformes | Gadidae | *Melanogrammus aeglefinus* (Linnaeus, 1758) | 96% EtOH | KJ193808 | X |  |
| 0874 | Vertebrata | Actinopterygii | Gadiformes | Merlucciidae | *Merluccius merluccius* (Linnaeus, 1758) | 96% EtOH | KJ193832 | X |  |
| 0649 | Vertebrata | Actinopterygii | Osmeriformes | Argentinidae | *Argentina sphyraena* Linnaeus, 1758 | 96% EtOH | KJ193815 | X |  |
| 0293 | Vertebrata | Actinopterygii | Perciformes | Gobiidae | *Pomatoschistus* sp. Gill, 1863 | 96% EtOH | KJ193838 | X |  |
| 0323 | Vertebrata | Actinopterygii | Perciformes | Gobiidae | *Crystallogobius linearis* (Düben, 1845) | 96% EtOH | KJ193816 | X |  |
| 0487 | Vertebrata | Actinopterygii | Perciformes | Carangidae | *Trachurus trachurus* (Linnaeus, 1758) | 96% EtOH | KJ193836 | X |  |
| 0273 | Vertebrata | Actinopterygii | Clupeiformes | Clupeidae | *Clupea harengus* Linnaeus, 1758 | 96% EtOH | KJ193828 | X |  |
| 0641 | Vertebrata | Actinopterygii | Pleuronectiformes | Pleuronectidae | *Hippoglossoides platessoides* (Fabricius, 1780) | 96% EtOH | KJ193817 | X |  |
| 1252 | Vertebrata | Actinopterygii | Pleuronectiformes | Pleuronectidae | *Kareius bicoloratus* (Basilewsky, 1855) | 96% EtOH | KJ193831 | X |  |
| 0902 | Vertebrata | Actinopterygii | Pleuronectiformes | Pleuronectidae | *Pleuronectes platessa* Linnaeus, 1758 | 96% EtOH | KJ193827 | X |  |
| 0761 | Vertebrata | Actinopterygii | Pleuronectiformes | Pleuronectidae | *Limanda limanda* (Linnaeus, 1758) | 96% EtOH | KJ193829 | X |  |
| 0605 | Vertebrata | Actinopterygii | Pleuronectiformes | Bothidae | *Arnoglossus laterna* (Walbaum, 1792) | 96% EtOH | KJ193826 | X |  |
| 0617 | Vertebrata | Actinopterygii | Pleuronectiformes | Soleidae | *Buglossidium luteum* (Risso, 1810) | 96% EtOH | KJ193814 | X |  |
| 0620 | Vertebrata | Actinopterygii | Scorpaeniformes | Cottidae | *Myoxocephalus scorpius* (Linnaeus, 1758) | 96% EtOH | KJ193809 | X |  |
| **Total** |  |  |  |  |  |  |  | **118** | **37** |
